# Sizing of nursing staff associated with self-care promotion in a pediatric semi-intensive care unit

**DOI:** 10.5935/0103-507X.20170027

**Published:** 2017

**Authors:** Armando dos Santos Trettene, Cassiana Mendes Bertoncello Fontes, Ana Paula Ribeiro Razera, Priscila Capelato Prado, Gesiane Cristina Bom, Lilia Maria von Kostrisch

**Affiliations:** 1 Programa de Pós-Graduação em Ciências da Reabilitação, Hospital de Reabilitação de Anomalias Craniofaciais, Universidade de São Paulo - Bauru (SP), Brasil.; 2 Departamento de Enfermagem, Universidade Estadual Paulista "Júlio de Mesquita Filho" - Botucatu (SP), Brasil.

**Keywords:** Self-care, Nursing workload, Staff sizing, Subacute care

## Abstract

**Objectives:**

To calculate and compare the nursing staff size associated with self-care promotion at a pediatric semi-intensive care unit.

**Methods:**

This was a prospective study in which 31 children and their caregivers participated. The nursing workload associated with each participant was evaluated at two different times (first and second hospital stays) using the Nursing Activities Score instrument. The first hospital stay corresponded to self-care promotion. Staff size was calculated according to the nursing hours recommended by the Nursing Activities Score instrument and by *Conselho Federal de Enfermagem* (COFEN) resolution no. 527/16, in the two hospital stays, and the results were compared.

**Results:**

The nursing workload in the first hospital stay (14.6 hours) was higher than the nursing workload in the second stay (9.9 hours) (p < 0.001). The Nursing Activities Score revealed that according to the nursing hours, the nursing staff size corresponded to 26 and 18 professionals in the first and second hospital stays, respectively, and to 15 professionals according to COFEN resolution no. 527/16.

**Conclusion:**

The number of personnel responsible for promoting self-care in pediatric semi-intensive care units, according to the nursing hours suggested by the Nursing Activities Score, was higher than that recommended by the existing legislation. This demonstrates the necessity of reconsidering staff size for this healthcare profile.

## Abstract (pt)

**Objetivos:**

Calcular e comparar o dimensionamento de pessoal no que diz respeito à promoção do autocuidado em unidade de terapia semi-intensiva pediátrica.

**Métodos:**

Estudo prospectivo, do qual participaram 31 crianças e seus respectivos cuidadores. Os participantes foram avaliados em dois momentos (primeira e segunda internação) quanto à carga de trabalho de enfermagem, por meio do *Nursing Activities Score*. A primeira internação correspondeu à promoção do autocuidado. O dimensionamento de pessoal foi calculado segundo as horas do *Nursing Activities Score* e a resolução do Conselho Federal de Enfermagem (COFEN) 527/16, nas duas internações, e estes dados foram posteriormente comparados.

**Resultados:**

A carga de trabalho de enfermagem na primeira internação (14,6 horas) foi maior em comparação à segunda (9,9 horas) (p < 0,001). O dimensionamento de pessoal segundo as horas, de acordo com o *Nursing Activities Score*, foi de 26 e 18 profissionais, e de 15 profissionais, conforme a resolução do COFEN 527/16.

**Conclusão:**

O quantitativo de profissionais referente à promoção do autocuidado em unidade de terapia semi-intensiva pediátrica, segundo as horas do *Nursing Activities Score*, foi superior ao recomendado pela legislação existente, demonstrando a necessidade de se reconsiderar o dimensionamento de pessoal neste perfil assistencial.

## INTRODUCTION

The efficient use of resources is widely discussed as a method to reduce costs in institutions providing health care.^(1)^ In this context, human and material resources are affected, in particular nursing staff, as they represent the majority of hospital personnel.^(2)^

Currently, the media often point out problems associated with health care, but the work overload and unhealthy conditions to which health care staff are exposed are rarely discussed.^(2)^

To provide care that considers different care scenarios and takes into account the quantitative and qualitative factors related to nursing staff, it is necessary to commit to staff sizing so as to adjust to care needs, avoid work overload and strengthen the safety culture for patients and staff.^(3)^

Staff sizing is defined as "the initial step in the staffing process aimed at predicting the number of workers per category necessary to address the needs of nursing care directly or indirectly delivered to the client". In the process of staff sizing, a number of variables, including nursing workload, are considered, and these must be measured according to the care profile and the intended standard of care.^(4)^

A variety of instruments, including the Nursing Activities Score (NAS), are used to measure nursing workload. Although this instrument was originally developed for critical care units, it is often used in other care profiles.^(5-9)^

The NAS directly expresses the percentage of time spent by nursing staff in delivering care to patients over a 24-hour period, with this percentage reaching a potential maximum of 176.8%. In addition to direct care activities, the score includes indirect care activities such as providing support and care to relatives and performing administrative and managerial tasks.^(5)^

The NAS, which was translated into Portuguese and adapted to the Brazilian context, consists of 23 items distributed among seven major categories: basic activities, ventilatory support, cardiovascular support, renal support, neurologic support, metabolic support and specific interventions.^(5,10)^

Oversized nursing staffs result in high costs to institutions and healthcare systems. In contrast, undersized staffs lead to work overload, occupational disease, absenteeism, dissatisfaction among personnel and clients, decreased quality of care, dehumanization and increased numbers of adverse events. These factors jeopardize patient safety and increase costs.^(11)^ Additionally, a reduced staff is essentially unavailable to provide emotional support to patients and relatives, especially in regard to pediatric patient care.^(12)^

The literature provides evidence of the benefits of an adequate quantitative and qualitative distribution of the nursing staff in various settings; these benefits extend beyond quality of care and involve the entire working process.^(13-15)^ The costs of negative events and outcomes of care delivery are generally higher than the corresponding operational costs required to avoid these events.^(16)^

In the absence of a widely accepted and available methodological benchmark, the *Conselho Federal de Enfermagem* (COFEN) adopted a resolution setting minimum parameters regarding staff sizing that is based on the complexity profile of patients. This resolution is based on several studies and was updated recently. In the updated resolution, nursing workload is defined according to patient care: minimal care, intermediate care, high-dependency care, and semi-intensive and intensive care.^(17)^

A number of studies have noted discrepancies between the nursing staff size recommended by the legislation and what is in fact necessary for each type of care.^(8,18,19)^ Therefore, it is clear that staff sizing is a great challenge.^(20)^

In this context, comparison of the methods used for staff sizing, as well as control and mastery of the methods used, is imperative. The arguments of management to administrators regarding adequate staffing must be well-founded, and the medium-term and long-term benefits of any proposed changes should be emphasized.^(21)^

Patients and relatives have different care profiles. Self-care is defined as "the performance or practice of activities that individuals initiate and perform on their own behalf to maintain life, health and well-being." Although adults may often care for themselves, infants, children, and, in some situations, the elderly, require care. In these cases, self-care depends on a provider, the self-care agent.^(22)^

Cleft lip and palate are described as the most prevalent facial anomalies. They can lead to functional, esthetic and psychosocial changes that require interdisciplinary care from birth, and this care must be delivered in centers of excellence.^(23)^ The level of care required is especially high in cases in which the anomaly is associated with genetic and clinical syndromes; such care often includes the use of feeding tubes, other techniques that facilitate feeding, and ventilation or respiratory equipment and justifies hospital stays in semi-intensive care units.^(8)^ However, these patients do not need to be hospitalized during treatment if their caregivers are trained to deliver care at the patient's home.^(24)^

Thus, the nursing care of children with cleft lip and palate, particularly in cases associated with genetic and clinical syndromes, consists largely of providing and maintaining oxygenation, feeding, hygiene and comfort. These children frequently use nasopharyngeal cannulae and feeding tubes. It is also necessary to train their caregivers to promote self-care; such training includes teaching, supervising and evaluating the care provided to the children.^(8,24)^

Managers and administrators tend to classify education and health guidance systems as minimal care because no sophisticated equipment or advanced technology is required to deliver the care provided by these systems.^(25)^ However, this care profile requires personnel who possess communication skills, who are available for and capable of teaching and evaluating, and who adopt a friendly attitude. These skills take time and professional competence to acquire; hence, they affect the nursing workload and, consequently, staff sizing.^(8,24,26)^

Although studies of staff sizing have been conducted, the literature regarding nursing work demands in semi-intensive care units is still very limited, particularly as regards pediatric units, where self-care is promoted through caregiver training.

Based on the above-mentioned considerations, what is the influence of self-care promotion on nursing staff sizing in a pediatric semi-intensive care unit?

We hope that this study will contribute to the process of staff sizing, considering its influence on the quality of service, professional satisfaction and patient safety. Additionally, we expect that the knowledge presented here will sensitize managers to issues related to human resource management.

Thus, the objective of this study was to calculate and compare staff sizing regarding self-care promotion in a pediatric semi-intensive care unit.

## METHODS

A prospective study was conducted at a medium-sized tertiary public hospital with 91 beds; this hospital represents a national and international model in the care of patients with craniofacial anomalies and related syndromes.

The hospital unit studied is a pediatric semi-intensive care unit; the unit contains eight beds, with one for the care of children between one day and two years old with cleft lip and palate and associated syndromes. The parents or caregivers typically stay at the unit for an entire morning to be trained in the care that must be provided at home after the patient is discharged from the hospital. Nursing care is guided by the Self-care Theory,^(22)^ among others.

An interdisciplinary staff was employed at the hospital; the staff included nurses, nurse technicians, physicians, physiotherapists, nutritionists, speech-language pathologists and occupational therapists. The nursing staff comprised one nursing coordinator, four nursing assistants, and eight nurse technicians, each of whom worked six hours per day and 36 hours per week.

Children diagnosed with Robin sequence represented 80% of the hospitalizations. Robin sequence is characterized by a triad comprising micrognathia, glossoptosis and cleft palate in most cases. This syndrome can appear in isolation (isolated Robin sequence) or in association with clinical syndromes and malformations.^(26)^ It usually leads to breathing and feeding difficulties. Treatment strategies include nasopharyngeal intubation, nasogastric tube feeding and the use of feeding facilitation techniques.^(8)^

The sample consisted of 31 hospitalized children and their respective caregivers from February to October 2014. Participation in the study was the inclusion criterion used for the caregivers. For the children, the inclusion criteria were hospital stay longer than 24 hours; accompanied by a caregiver; and use of nasopharyngeal intubation, nasogastric tube and/or feeding facilitation techniques. Children with Robin sequence associated with clinical syndromes and/or comorbidities were excluded from the sample.

The study was initiated after approval by the Human Research Ethics Committee of the *Hospital de Reabilitação de Anomalias Craniofaciais* of the *Universidade de São Paulo*, opinion no. 512376 and CAAE: 25895513900005441. All participants gave written informed consent to participate in the study, in compliance with resolution no. 466/12.

The children and their caregivers were evaluated according to nursing workload at two different times, during the first and second hospital stays. During the first hospital stay, the caregivers were trained by the nursing staff to take care of the children with isolated Robin sequence, i.e., the nursing staff promoted self-care. It is important to note that the criteria for hospital discharge included caregiver training in home care.

In the second hospital stay, the children were evaluated as to their clinical progression, i.e., nasopharyngeal intubation removal and oral feeding progression. Although the caregivers were already qualified to take care of the children, they needed the services provided by the nursing staff because of the changes in the children's treatment.

To evaluate the nursing workload, the NAS was used every 24 hours. All information listed in the shift reports and medical records of the patients was analyzed.

Items 7a and 7b of the NAS are considered to refer to self-care promotion because they comprise support and care provided to the relatives, including training of the relatives in self-care. Item 7a addresses the support and care provided to the relatives of patients who need exclusive dedication for approximately one hour during a shift, whereas item 7b is scored in cases in which the support and care provided to the relatives of patients who require exclusive dedication requires three or more hours during a shift.^(5)^

The data were collected by the researcher with the help of two nurses who worked in the semi-intensive care unit and were trained to apply the NAS but did not know the purpose of this study. In addition, a tutorial for data collection was prepared and validated, in compliance with the recommendations of the NAS's authors.^(5)^ This approach improved the reliability of the data collection because the time spent on indirect care of the patient and their relatives and/or informal caregivers was usually not recorded in the medical reports or visiting nursing records.

First, the nursing workload in the first and second hospital stays was evaluated. The two workloads were then compared to identify significant differences, focusing on the items corresponding to support and care provided to relatives because these items included interventions related to caregiver training in the care of children with isolated Robin sequence.

Next, the number of hours of nursing care recommended by the NAS for each of the two hospital stays were compared to each other and to the hours recommended by COFEN resolution no. 527/16 for patients under semi-intensive care.^(17)^

To make it possible to compare the nursing workload according to the hours recommended by the NAS and by COFEN resolution no. 527/16 according to the type of care the patient needed, it was necessary to transform the scores into hours.^(17)^ For this purpose, each single unit of the NAS score was considered to be equal to 0.24 hours.^(5)^

To calculate the daily nursing staff size according to the hours recommended by the NAS, the following equation,^(18)^ assuming a six-hour workday and a productivity (effective workday) of 80%, was used:

$$Q_{\mathrm{daily}}=\frac{n\;x\;\sum \mathrm{NAS}}{t\;x\;p}$$

Where: ***Q_daily_ =*** daily number of nursing personnel, ***n* =** mean number of patients per day, ***∑ NAS =*** mean daily hours recommended by NAS, *t =* length of the workday (6 hours), and ***p =*** productivity (0.80).

To calculate the annual nursing staff size according to the hours recommended by the NAS, the following equation was used. The technical safety index (TSI), which corresponded to the absences due to days off (weekly paid break and holidays not falling on Sundays - 0.19 or 19%) was considered, in addition to the TSI proposed to cover vacations and unexpected absences (0.15 or 15%). The final TSI was 1.34 (34%).^(17)^

$$Q_{\mathrm{annual}}=Q_{\mathrm{daily}}\;x\;\mathrm{TSI}$$

Where: ***Q_annual_ =*** annual number of nursing personnel, ***Q_daily_ =*** daily number of nursing personnel, and ***TSI* =** Technical Safety Index (1.34).

To calculate the nursing staff size according to COFEN resolution no. 527/16, the number of nursing hours was first obtained.^(17)^ The nursing hours corresponding to semi-intensive care (10 hours), an occupation rate of 80%, and the Marinho constant corresponding to a 36-hour workweek (0.2236) were considered. The following equation was used:

$$\begin{array}{c} \mathrm{TNH}=\mathrm{OR}\;x\;\mathrm{NH}\\ \mathrm{NP}=\mathrm{MK}\;x\;\mathrm{TNH} \end{array}$$

Where: ***TNH =*** total nursing hours, ***NH =*** nursing hours (10 hours), ***OR =*** occupation rate (0.80), ***NP =*** number of personnel, and ***MK =*** Marinho constant (0.2236).

Student's *t*-test was used for the statistical analysis. P-values ≤ 0.05 (5%) were considered to indicate statistically significant differences.

## RESULTS

Thirty-one children with isolated Robin sequence and their respective caregivers participated in the study. The mean age of the children was 26 days (standard deviation [SD] = 16.9 days), ranging from 6 to 64 days, with a predominance of females (68%). Most of the caregivers (97%) were the mothers of the children. The mean age of the caregivers was 24.7 years (SD = 5.4 years); 74% were married, 71% had an only child, 61% had a low socioeconomic status, and 40% had finished high school.

Five hundred and nineteen evaluations were performed; 320 of these were related to the first hospital stay, and 199 were related to the second hospital stay. The mean duration of the first hospital stay was 10.3 days (SD = 5.7 days), and the mean duration of the second hospital stay was 6.4 days (SD = 3.4 days).

The mean nursing workload during the first hospital stay was 60.9% (SD = 12.8%), whereas the mean nursing workload during the second hospital stay was 41.6% (SD = 7.3%) (Table 1). The nursing workloads during the first and the second hospital stays were significantly different (p < 0.001) (Table 2).

**Table 1:** Nursing workload during the first and second hospital stays

Characteristics	Mean	Standard deviation	Maximum	Minimum	Median
NWL in the first hospital stay	60.9	12.8	89.5	38.9	57.8
NWL in the second hospital stay	41.6	7.3	63.2	30.3	40.2
Difference	19.3	5.5	26.3	8.6	17.6

NWL - nursing workload.

**Table 2:** Comparison of the mean Nursing Activities Score between the two hospital stays

Characteristics	Mean	Standard deviation	p value
NAS in the first hospital stay	60.9	12.8	
NAS in the second hospital stay	41.6	7.3	< 0.001*
Difference	19.3	13.2	

NAS - Nursing Activities Score. Student’s *t*-test.

* Statistical significance (p ≤ 0.05).

Evaluation of the prevalence of the interventions corresponding to the NAS items during the first hospital stay showed a predominance of the following items: monitoring and control (100%), hygiene procedures (100%), medication, vasoactive drugs excluded (100%), mobilization and positioning (100%), support and care provided to relatives (100%), care of artificial airways (100%) and enteral feeding through a gastric tube or another gastrointestinal route (100%) (Table 3). With respect to the prevalence of the interventions corresponding to the NAS items during the second hospital stay, the following items were predominant: monitoring and control (100%), hygiene procedures (100%), support and care provided to relatives (100%) and medication, vasoactive drugs excluded (100%) (Table 3).

**Table 3:** Distribution of the therapeutic interventions of the Nursing Activities Score according to its individual items in the two hospital stays

Nursing Activities Score items	First hospital stay (%)	Second hospital stay (%)
1. Monitoring and control	100	100
1a. Hourly vital signs, calculation and regular registration of fluid balance	62	90
1b. Personnel at bedside and observation or continuous activity for 2 hours or more during any work shift (6 hours) due to safety, severity or therapy needs	37	10
1c. Personnel at bedside and observation or continuous activity for 4 hours or more at any work shift (6 hours) due to safety, severity or therapy needs	1	-
2. Laboratory (biochemical and microbiological) investigations	21	20
3. Medication (vasoactive drugs excluded)	100	100
4. Hygiene procedures	100	100
4a. Continuous or intermittent performance of hygiene procedures requiring less than 2 hours per nursing shift (6 hours)	69	96
4b. Continuous or intermittent performance of frequent hygiene procedures, i.e., from three to four times per nursing shift (6 hours), of frequent hygiene procedures requiring less than 2 hours per nursing shift or care according to item 4 for children who are under contact precautions	31	4
6. Mobilization and positioning	100	100
6a. Performance of mobilization and positioning procedures from three to six times per nursing shift (6 hours) for which only one nursing staff member is needed	76	90
6b. Performance of mobilization and positioning procedures more than six times per nursing shift (6 hours) for which only one nursing staff member is needed	24	7
7. Support and care provided to relatives	100	100
7a. Support and care provided to relatives and patients requiring full dedication for up to 1 hour (continuously or with interruptions)	54	80
7b. Support and care provided to relatives and patients requiring full dedication for more than 1 hour (continuously or with interruptions)	46	20
8. Routine administrative and managerial tasks	94	93
8a. Performance of routine administrative and managerial tasks	69	80
8b. Performance of routine administrative and managerial tasks requiring full dedication for approximately 1 to 2 hours (continuously or with interruptions) in a nursing shift	25	13
9. Ventilatory support	16	2
10. Care of artificial airways	100	55
11. Treatment for improving lung function	26	8
15. Cardiopulmonary resuscitation	1	-
21. Enteral feeding through gastric tube or other gastrointestinal route	100	90
22. Specific interventions in the intensive care unit	15	5
23. Specific interventions outside the intensive care unit	25	17

Regarding the NAS items regarding activities related to self-care promotion (items 7a and 7b - support and care provided to relatives), the comparison between the first and second hospital stays showed a significant difference in the NAS scores (p < 0.001) (Table 4).

**Table 4:** Comparison of the mean Nursing Activities Score between the two hospital stays for items related to caregiver training

Variable	Mean (%)	Standard deviation (%)	p value
Item 7a in the first hospital stay	53.9	13.8	
Item 7a in the second hospital stay	79.5	18.2	< 0.001*
Difference	-25.6	22.5	
Item 7b in the first hospital stay	46.1	13.8	
Item 7b in the second hospital stay	20.5	18.2	< 0.001*
Difference	25.6	22.5	

Student’s *t*-test.

* Statistical significance (p ≤ 0.05).

Considering that each unit in the NAS is equivalent to 0.24 hours and that, on average, the measurements of the first and second hospital stays were 60.9% and 41.6%, respectively, 14.6 productive nursing hours were expended during the first hospital stay, and 9.9 productive nursing hours were expended during the second hospital stay in a 24-hour period.

According to the hours recommended by the NAS, the daily staff size during the first hospital stay (self-care promotion) was 16 professionals, whereas the annual staff size was 26 professionals, comprising 11 (42%) nurses and 15 (58%) nurse technicians.^(17)^ It is important to note that this staff size is related to 24 hours of nursing assistance, i.e., the staff must be divided into work shifts corresponding to 24 hours. The nursing workload during the 24-hour shift was 93.4 hours.

According to the hours recommended by the NAS, the daily staff size during the second hospital stay was 14 professionals, whereas the annual staff size was 18 professionals, comprising 8 (42%) nurses and 10 (58%) nurse technicians.^(17)^ It is important to reiterate that the staff must be divided into work shifts corresponding to 24 hours. The nursing workload during the 24-hour shift was 63.4 hours.

The calculation of the staff size according to COFEN resolution no. 527/16 considered an occupation rate of 80%, the number of nursing hours for patients receiving semi-intensive care (10 nursing hours), and a Marinho constant corresponding to 36 hours per week (0.2236).^(17)^ The result was 15 professionals, including 6 (42%) nurses and 9 (58%) nurse technicians. The nursing workload during the 24-hour period was 64 hours.

**Figure 1:**
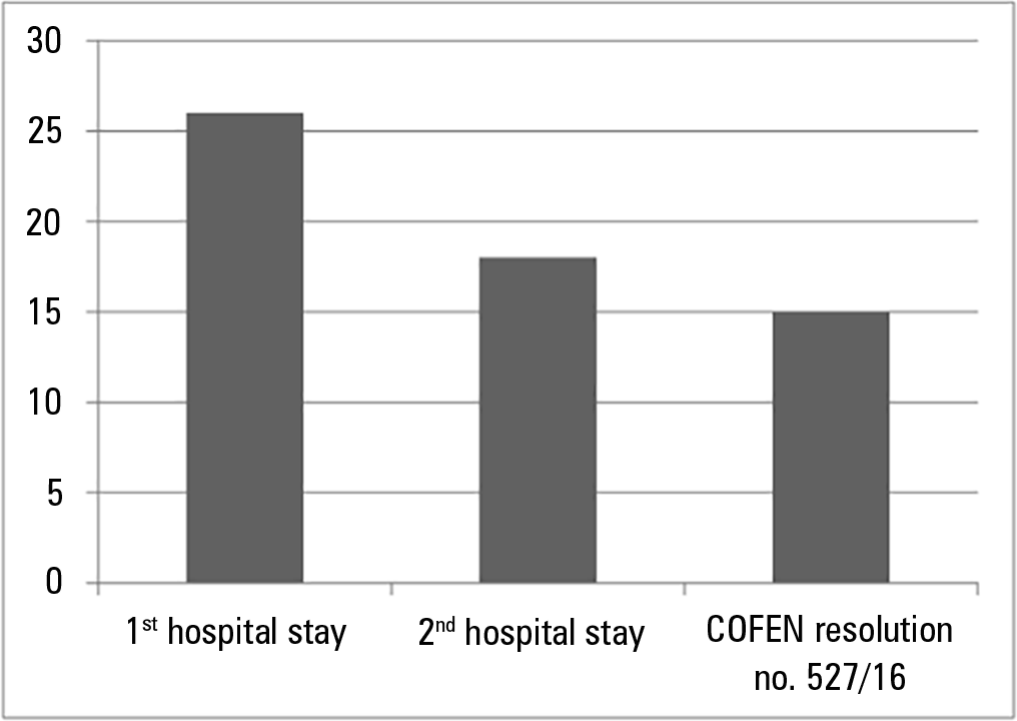
Differences between nursing staff sizing according to the hours recommended by the Nursing Activities Score instrument, in the two hospital stays, and to *Conselho Federal de Enfermagem* resolution 527/16. COFEN - *Conselho Federal de Enfermagem*.

The staff size during self-care promotion was higher than the staff size during the second hospital stay and higher than that calculated according to COFEN resolution no. 527/16^(17)^ (Figure 1).

## DISCUSSION

The comparison of nursing workload according to the hours recommended by the NAS tool and COFEN resolution No. 527/16^(17)^ showed that the nursing workload recommended by the NAS was higher, corroborating the findings reported in the literature.^(8,18,19,27)^ A previous study that evaluated nursing workload in a specialized semi-intensive care unit showed that nursing workload calculated according to the NAS was higher than that recommended by the COFEN resolution, which consequently affected staff size.^(8)^

Another study that evaluated staff size in an adult semi-intensive care unit according to the number of hours recommended by the NAS in comparison to the number of hours recommended by COFEN showed that the staff size was lower than the recommended size.^(27)^

A third study conducted in different sectors of a neonatal care unit compared the number of personnel according to the NAS and the current legislation and concluded that there is a significant personnel shortage, i.e., the staff size recommended by the NAS was higher.^(18)^

A study conducted at an inpatient unit using the NAS concluded that the nursing hours required corresponded to semi-intensive and intensive care patient profiles and indicated an excessive nursing workload.^(19)^

In contrast, another study found that the workload measured by application of the NAS was lighter than the workload recommended by COFEN. The latter result may be associated with the presence of low-complexity patients, as there was no semi-intensive care unit at that institution.^(28)^

In addition, another study analyzed the nursing staff size at an adult intensive care unit by applying the NAS and following the COFEN recommendation and concluded that, despite some limitations, both methods are efficient for calculating staff size.^(10)^

In view of the foregoing, it is worth noting that COFEN has recently updated its patient classification as well as the nursing hours for each care profile. The nursing hours provided to semi-intensive care patients, for example, were increased from 9.4 to 10 hours.^(17)^ In this sense, the present study is pioneering in the use of these recommendations.

The evaluation of nursing workload associated with specific working processes is considered an important method for defining staff size, the distribution of work according to various professional categories and the measurement of the care required by each type of client.^(15,29,30)^

However, it is important to emphasize that staff size alone does not necessarily indicate the quality of care; thus, the qualitative aspects of each situation should also be analyzed. In this context, COFEN states that the nursing staff necessary to provide care to semi-intensive patients should consist of 42% nurses and 58% nurse technicians.^(17)^

This recommendation assumes that nurses have specialized scientific knowledge, which means that not following this recommendation would result in an inferior quality of care. However, in practice, it is observed that this rule is not strictly followed, mostly due to unfavorable socioeconomic conditions and public policies.

A study aimed at evaluating the parameters set forth by COFEN that is considered a Brazilian model for nurse staff sizing demonstrated the benefits of the values of the average care time at adult ICUs. However, it showed that the recommendations regarding the professional categories are far from the reality, especially with respect to the number of nurses.^(20)^

The current severe fiscal crisis in Brazil has directly resulted in restrictions to the health system, including the supply of nursing human resources. In turn, nurse staffing has a direct effect on the work process and has repercussions regarding the quality of care and, consequently, on patient safety.^(31,32)^

The benefits of a quantitatively and qualitatively adequate nursing staff are related to a lower incidence of iatrogenic diseases and adverse events, healthcare-related infections, hospital readmissions, and mortality, among others.^(33-39)^

It is of fundamental importance for the improvement of patient safety that healthcare managers invest in human resources, as they are considered the primary determinant of the quality of care. A recent study aimed at identifying the main factors related to the intervention by nursing staff in patient safety stressed this necessity.^(40)^

Provision of the appropriate nursing staff size provides benefits that extend beyond those related to patient safety, including issues related to professional satisfaction and the delivery of humanistic and holistic care in addition to institutional repercussions; this is because nursing staff size directly affects quality, which is highly important and desirable. Quantitative and qualitative staff sizing is a huge challenge; however, we will only be able to overcome this challenge by applying scientific methods.^(20)^

Finally, it is important to mention the limitations of this study. Because the specificity of our working process is linked to the profiles of the patients treated, no generalization of the results is possible. Thus, it is necessary to conduct studies that compare the use of different nursing staff sizing methods with the use of the methods recommended by government agencies for different care profiles.

In addition, although the present study sought to increase methodological rigor by training the personnel involved in the study, creating and validating a tutorial, and including nurse assistants in data collection, the subjective scoring of the items listed in the NAS by different nurses can be considered a limitation.

From the results reported here, it is evident that it is necessary to identify the specific care needs of the users within each care unit and not simply follow the recommendations of the government agencies. Thus, the main contribution of this study is the identification of the need to re-evaluate the nursing staff size required for self-care promotion at a pediatric semi-intensive care unit.

## CONCLUSION

The nursing staff size needed for self-care promotion at a pediatric semi-intensive care unit based on the hours recommended by the Nursing Activities Score was higher than that recommended by current legislation. This shows that it is necessary to reconsider the staff size for this care profile.
